# Metabolic adaptation of microbial communities to ammonium stress in a high solid anaerobic digester with dewatered sludge

**DOI:** 10.1038/srep28193

**Published:** 2016-06-17

**Authors:** Xiaohu Dai, Han Yan, Ning Li, Jin He, Yueling Ding, Lingling Dai, Bin Dong

**Affiliations:** 1 State Key Laboratory of Pollution Control and Resource Reuse, College of Environmental Science and Engineering, Tongji University, 1239 Siping Road, Shanghai 200092, PR China

## Abstract

A high solid digester with dewatered sludge was operated for 110 days to ascertain the interactions between bacterial and archaeal communities under ammonium stress, as well as the corresponding changes in bio-degradation mechanisms. The volatile solids reduction (95% confidence intervals in mean) changed from 31.6 ± 0.9% in the stable period (day 40–55) to 21.3 ± 1.5% in the last period (day 71–110) when ammonium concentration was elevated to be within 5,000–6,000 mgN/L. Biogas yield dropped accordingly from 11.9 ± 0.3 to 10.4 ± 0.2 L/d and carbon dioxide increased simultaneously from 35.2% to 44.8%. *Anaerobranca* better adapted to the ammonium stress, while the initially dominant protein-degrading microbes-*Tepidimicrobium and Proteiniborus* were suppressed, probably responsible for the increase of protein content in digestate. Meanwhile, *Methanosarcina*, as the dominant Archaea, was resistant to ammonium stress with the constant relative abundance of more than 92% during the whole operation. Nonmetric Multidimensional Scaling (NMDS) analysis was thus conducted which indicated that the gradually increased TAN dictated the bacterial clusters. The dominant *Methanosarcina* and the increased carbon dioxide content under ammonium stress suggested that, rather than the commonly acknowledged syntrophic acetate oxidation (SAO) with hydrogenotrophic methanogenesis, only SAO pathway was enhanced during the initial ‘ammonium inhibition’.

The anaerobic digestion process, which can degrade and stabilize organic substances while simultaneously generating valuable energy source - biogas, is considered a sustainable treatment technology for sewage sludge^1^. Recently, due to advantages including higher organic loading rate, lower energy requirement for heating, and smaller reactor volume, high solid anaerobic digestion processes (with total solids input over 10%) have been widely implemented and are claimed to outperform traditional low solid anaerobic digestion processes^2^. However, the stable and optimal performance of high solid anaerobic digestion processes is easily impacted by operational parameters and accumulating chemicals^3^,^4^.

In general, ammonia is a necessary nutrient for the growth of microorganisms, playing significant roles in maintaining the required alkalinity and stability in anaerobic digestion systems^5^,^6^. Ammonia present in digesters is not only derived from the feedstocks, but also from the breakdown of proteins, urea, and nucleic acids^7^. High strength ammonia, in the form of either total ammonium nitrogen (TAN) or free ammonia nitrogen (FAN), is widely considered to be one of the causes of digester failure due to inhibition of microbial activities^3^,^6^,^8^. FAN is mainly dependent on TAN, pH, and temperature, and is regarded freely membrane-permeable^3^. When FAN diffuses into microbial cells, it can disturb the balance of intracellular pH, leading to lower enzymatic activities and abnormal material transportation^9^,^10^. Subsequently, FAN concentration of 700–1,100 mgN/L was shown to be capable of triggering inhibition in many kinds of substrates^11^,^12^,^13^. On the contrary, studies supported that TAN concentrations are more accountable than FAN for the inhibition^14^,^15^. For example, Lay *et al*.^14^ stated that even though FAN concentrations reached up to 900 mgN/L at pH 9.0 when TAN was around 3,000 mgN/L, the performance of the mesophilic high solid sludge in a batch digester was not impaired. Generally, TAN exceeding 5,000 mgN/L would result in a sharp reduction in methane production^16^, such as a 27% decrease in biogas with a TAN concentration of 5,980 mgN/L^17^, a sharp reduction of methane production as TAN concentrations reached 5,500 mgN/L^16^, and a reduction of methanogenic activity by 50% with TAN of 5,500 mgN/L^14^. Furthermore, 80–90% of methane production was suppressed when TAN concentration reached 8,000 mgN/L^18^. To sum up, the inhibitory TAN concentration ranged widely, from 1,500–14,000 mgN/L^8^,^12^,^19^, which could be attributed to the effects related to inoculum origin, substrate properties, operational conditions (temperature, pH), acclimation periods, and anaerobic microbes^3^,^20^.

In particular, the reduction of biogas has been preferentially ascribed to the inhibition of methanogenesis, as which is assumed to be more readily suppressed by ammonium than hydrolysis and acidogenesis^21^,^22^. Sung and Liu^8^ reported that acetotrophic methanogens are more susceptible to ammonium stress than hydrogenotrophic ones. However, even under the condition of 7,000 mgN/L TAN in a batch digester operated for 29 days, acetoclastic methanogenesis was still found to be catalyzed by *Methanosarcina*, playing a primary role in methane yield^23^. When high solid anaerobic digestion systems are applied to treat protein-rich sewage sludge, ammonium concentration usually exceeds the tolerance of microbes degrading organic substances. As reported by Niu *et al*.^24^, the TAN thresholds for the inhibition of protein and carbohydrate degradation were 3,000 mgN/L and 4,000 mgN/L, respectively. Apart from significant inhibition, an ammonium-induced “inhibited steady-state” for an apparently stable but suboptimal process might occur in anaerobic digestion with feedstocks ranging from 2,800–4,570 mgN/L TAN^25^,^26^. Thus, the detailed metabolic adaptation of microbial communities under ammonium stress should be clearly understood, as an in-depth understanding of it will provide knowledge regarding the comprehensive manipulation of microbes to avoid the deterioration of digester performance.

The objective of the current study was to investigate the effects of elevated ammonium stress on both evolution of individual organic composition and relevant microbial adaptation in a long-term high solid anaerobic digester with dewatered sludge. The changes of organic matters such as proteins, carbohydrates, and lipids in anaerobic digestion were thus monitored. The dynamics of both bacterial and archaeal communities was analyzed by pyrosequencing and presented with changes in relative abundance. Furthermore, Nonmetric Multidimensional Scaling (NMDS) analysis was conducted to reveal the relationships between bacterial communities and the kinetics fitting parameters. Finally, combined with the analysis on the changes of *Methanosarcina* density before and after ammonium addition by real-time polymerase chain reaction, the understanding on the instinctual interactions responding to ammonium stress was subsequently obtained.

## Results

### Overall performance of the digester

**Figure 1:**
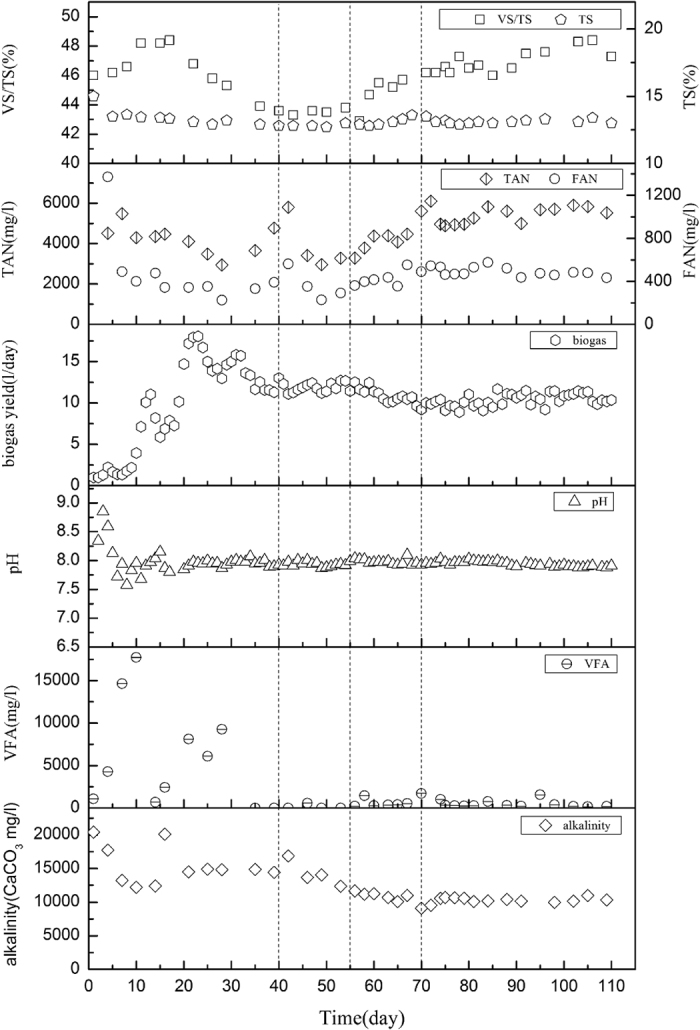
Time course of operational parameters during the operation.

The performance data for TS, VS/TS, TAN, FAN, biogas yield, pH, VFAs, and alkalinity is shown in Fig. 1. During the start-up period from day 1 to day 40, the digester was unstable with a sharp decrease of VS/TS in the slurry. The overall downward trend of VS/TS indicated the improved hydrolysis and degradation of organic matters. Biogas yield reached a climax of 18.0 L/d on day 23 with the accumulated VFA at a relatively high level, and then decreased steadily to a stable level of around 11.0 L/d. During this time, the VFA content decreased to less than 600 mg/L, indicating that a substrate competition balance was achieved between methanogens and acetogens. Meanwhile, pH dropped from the initial 8.85 to an average of 7.98 during the start-up period. Alkalinity was above 12,000 mg/L, ensuring the buffer capacity of the system. TAN concentration was 4,209 mgN/L on average (Table 1), which was already relatively high and could be attributed to the rapid hydrolysis of protein during the period. The variation of TAN during the start-up period could result from the dynamics between the utilization of ammonium by microbes for growth and reproduction and the generation from the breakdown of protein.

During the steady-state phase from day 41 to day 55, a stable performance was eventually achieved, with a biogas yield of 11.9 ± 0.3 L/d and no VFA accumulation. The pH value was constant at 7.94 ± 0.02. VS/TS maintained a relatively constant level of 43.6 ± 0.3% with a VS reduction of 31.6 ± 0.9%. During this period, TAN concentration was at a level of 3,860 ± 2,080 mgN/L. For unknown reasons, TAN concentration peaked at day 42, with a sudden rise to 5,800 mgN/L. In addition to this unexpected phenomenon, biogas yield increased from 11.1 L/d to 12.4 L/d since day 42 to 47, with alkalinity also increasing significantly on day 42. However, the process transited quickly from the fluctuation.

From day 56 on, ammonium concentration was progressively elevated by mixing ammonium chloride in the feedstock till it reached 4,287 ± 667 mgN/L by day 70, with FAN concentrations within the range of 429 ± 66 mgN/L. Accompanied by the increase of ammonium loading, pH was not obviously influenced, with a relatively constant value of 7.98 ± 0.03. However, a significant drop in biogas yield from 12.5 L/d on day 56 to 9.2 L/d on day 70 was observed with the simultaneous decrease of alkalinity from 11,674 to 9,106 mg/L. During this ammonium adjusting period, VS/TS dramatically increased and the VS reduction declined sharply from 31.6 ± 0.9% at the steady state to 27.3 ± 3.5%. In general, the shock ammonium loading deteriorated the operational performance.

In the last period, TAN concentrations maintained at the higher level (5,443 ± 247 mgN/L) than earlier stages and FAN was accordingly at 490 ± 25 mgN/L. pH was at 7.94 ± 0.01 without significant variation and alkalinity was also at a relatively constant level of 10,327 ± 229 mg/L, coupled without VFA accumulation. It was interesting that with high TAN concentration and a continuous elevation of VS/TS, the biogas yield did not present a significant correlation with VS reduction. From day 71 to day 85, biogas yield maintained at a lower constant level of 9.8 ± 0.3 L/d, then recovered slightly to 10.7 ± 0.3 L/d till day 110. The biogas yield recovered slightly from day 86 even with the elevation of VS/TS, which could be attributed to the enhanced CO_2_ production with the decrease of methane content from 64.8% in Phase II to 55.2% in Phase IV.

### Dynamics of macromolecular biodegradation

**Figure 2:**
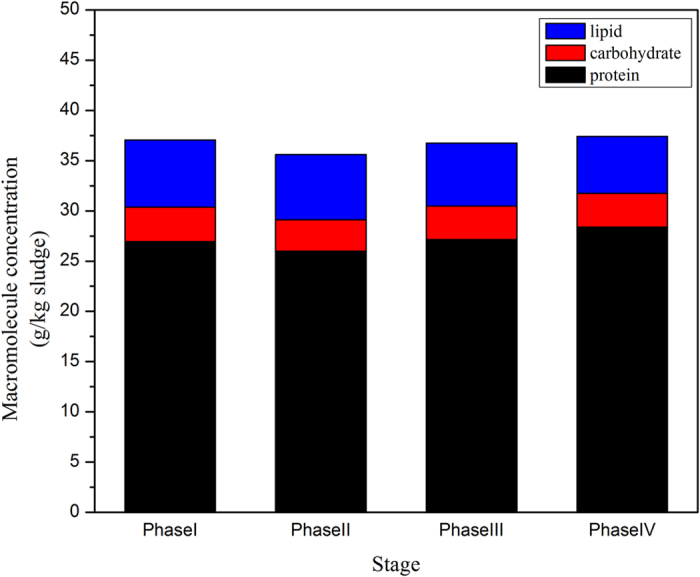
Summarized changes of protein, carbohydrate and lipid contents in digested sludge during the different operational phase.

Proteins, carbohydrates and lipids account for a large part of organic content in sewage sludge. Biogas is generated by the bioconversion of these compounds under anaerobic conditions. For instance, proteins are hydrolyzed to amino acids and further degraded to ammonium, VFAs, carbon dioxide, hydrogen gas, and reduced sulphur^27^. The particulate carbohydrates are finally degraded to VFAs through hydrolysates such as cellobiose and glucose^28^. Neutral fat, as the main component of lipids in sewage sludge, is generally hydrolyzed to long chain fatty acids and glycerol, which are ultimately degraded by H_2_-producing acetogenic bacteria via bio-oxidation^27^. The average value of the organic content including proteins, carbohydrates, and lipids in each stage during the 110-day operation is presented in Fig. 2. In the steady-state period of the digester from day 41 to 55 (Figure S1), the content of all the organic matter was constantly maintained. The respective degradation rate of proteins, carbohydrates and lipids was 35.1%, 39.9% and 2.1% at a relatively stable level, except for the slight decrease of remaining protein content on day 42. Ammonium, as the by-product of protein degradation, suddenly increased and coincided with the exception in protein content on the same day. The protein content increased along with the acute ammonium stress after day 56, indicating that an inhibition of protein hydrolysis was caused by the elevated TAN. Compared to the steady-state (Phase II), the protein degradation rate decreased by 8.2% in Phase III and 14.5% in Phase IV (p in ANOVA = 0.03). Carbohydrates and lipids showed no significant difference under acute ammonium stress. From day 71, the digester entered a period with relatively constant TAN concentration (5,000–6,000 mgN/L), during which carbohydrates and lipids maintained constant concentrations till the end of the operation, elucidating that microbes degrading carbohydrates and lipids could adapt to the acute and chronic ammonia stress.

### Responses of microbial communities to ammonium stress

**Figure 3:**
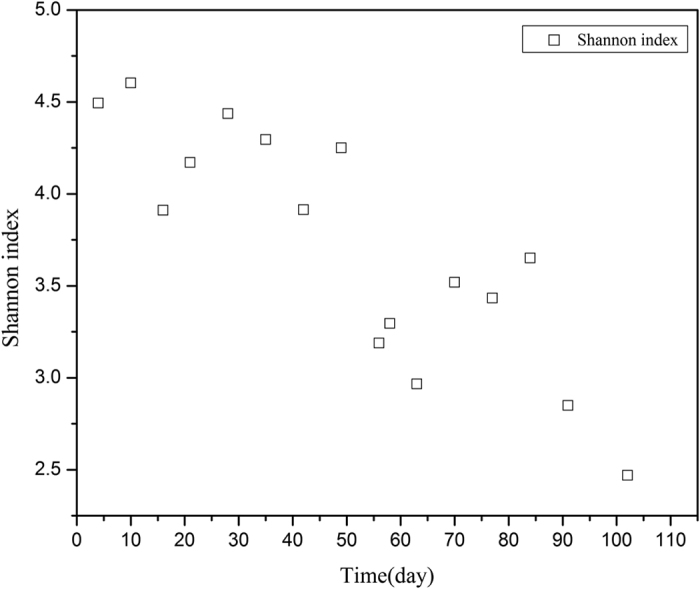
Evolution of Shannon index of bacterial communities during the operation.

In anaerobic digestion systems, bacteria are generally involved in hydrolysis and acetogenesis. To a large extent, the shift in bacterial communities responds to the imposed ammonium stress, and results in the evolution of macromolecular degradation. The bacterial diversity estimated from the 16 collected samples covering the four phases during the whole operation was presented by Shannon index (Fig. 3). Before the ammonium addition on day 56, Shannon index was around 4.3. It dramatically dropped to 3.2 right after the addition, and further achieved 2.5 at the end of the operation, which specified the adverse impact of high ammonium concentration on bacterial diversity during anaerobic digestion.

**Figure 4:**
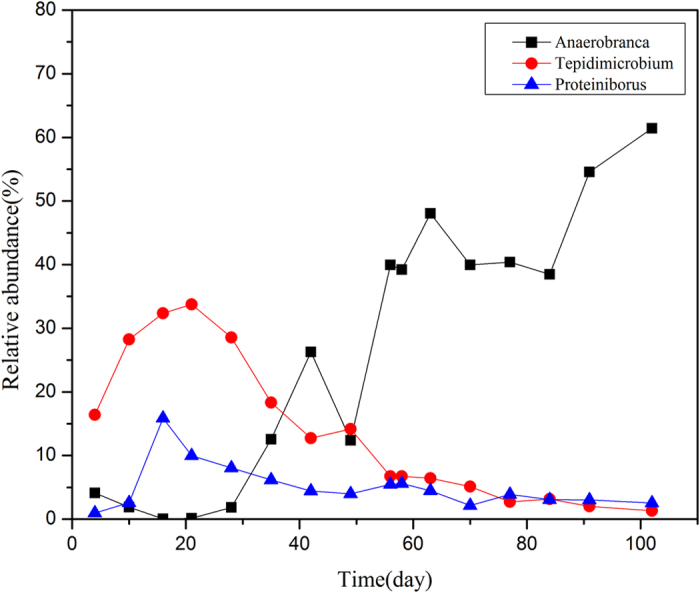
Variations of the three dominant bacteria during the operation.

The evolution of relative abundance of the dominant bacteria during the operation was shown in Fig. 4 at genus level. During the start-up period, *Tepidimicrobium* and *Proteiniborus* first rose to the climax of 33.8% on day 21 and 15.8% on day 16, respectively. Then, both of them dropped to 18.3% and 6.2% on day 35. Bacterial abundance gradually achieved stable in Phase II except for a sharp rise of *Anaerobranca* on day 42. Since ammonium was added from day 56, a positive link between *Anaerobranca* and TAN concentration was observed, while *Tepidimicrobium* and *Proteiniborus* showed totally opposite trend. Declines of the two kinds of bacteria indicated the inhibited protein hydrolysis triggered by the ammonium elevation from day 56.

**Figure 5:**
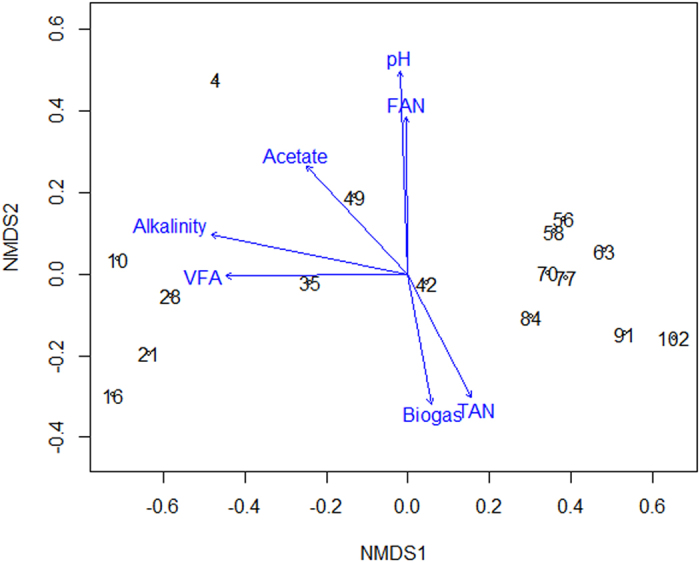
Joint-plot NMDS maps revealing the qualitative bacterial community shifts from the pyrosequencing results. Each community profile on the maps is labeled with a number indicating the operation time (day). Solid arrows project the correlations between a set of process parameters and each ordination axis.

The shift of the entire bacterial community (Figure S2) was further analyzed by incorporating the changes of operational parameters. The results from NMDS analysis (Fig. 5) indicated that the shift of the bacterial communities corresponded to the elevation of ammonium stress. The stress for the solution was lower than 0.05 and sufficient to reach the requirement of a reliable portrait of the changes in bacterial community structure^29^. The bacterial communities in the samples taken from the beginning of the study until day 55 were completely different from the bacterial communities in the samples taken after this point. Vectors representing environmental factors were extracted and grouped according to their correlations to the community profile, with coefficients higher than 0.25. The pH and FAN, contrary to the reported result by De Vrieze *et al*.^30^, did not show their contribution to the changes of the bacterial communities during the operation. Alkalinity and VFA were observed to be responsible for the stability in the bacterial communities before ammonium doses. TAN to some extent dictated the shift of the bacterial communities, which partially proved the results proposed by Nakakubo *et al*.^15^ that methane yield was significantly impacted by TAN with a high correlation coefficient (R^2^ = 0.91).

**Figure 6:**
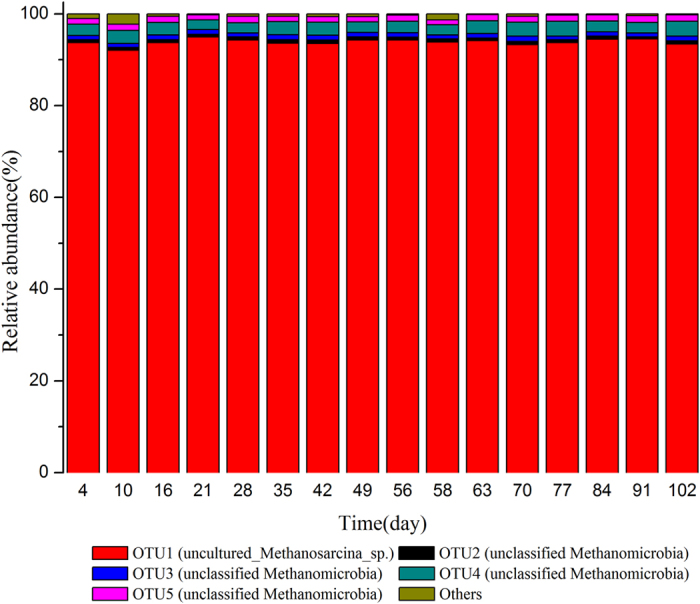
Changes of relative abundance of archaea at OTU level.

In total, there were 32 operational taxonomic units (OTUs) detected within archaeal communities. The five most dominant ones were selected and the remaining was grouped into others. Their changes were presented in Fig. 6. OTU1, accounting for more than 92% of the whole archaeal communities during the operation, was clustered to *Methanosarcina* which constantly played the key role in methane production even after ammonium addition from day 56. *Methanomicrobia*, participating in hydrogenotrophic methanogenesis^31^, was the sub-dominant in our digester. The quantitative PCR results of *Methanosarcina* (Fig. S3) further revealed that the average values in each stage were 1.28 × 10^6^ ± 0.64 × 10^6^ (Phase I), 3.43 × 10^6^ ± 2.91 × 10^6^ (Phase II), 2.98 × 10^6^ ± 1.22 × 10^6^ (Phase III), and 3.58 × 10^6^ ± 1.18 × 10^6^ copies/g sludge (Phase IV). The *Methanosarcina* density in Phase II increased comparing to the initial phase I, and the digester reached the stable state. The shock loading of ammonium on phase III indeed induced an acute inhibition on *Methanosarcina*. It was remarkable that the *Methanosarcina* recovered in phase IV with similar density in phase II even at TAN concentration of 5,000–6,000 mgN/L. The cultivation of *Methanosarcina* under high ammonium strength was thus achieved. In addition, no *Methanosarcina* shift at OTU level was observed in our study, which confirmed the acetoclastic methanogenesis dominated acetate utilization in the digester.

## Discussion

Niu *et al*.^24^ reported that carbohydrate degradation efficiency was 60% at 6,000 mgN/L TAN concentration, and stable protein conversion could be achieved only when TAN was lower than 3,000 mgN/L. In their study, the protein removal efficiency decreased from 40% at TAN of 1,500 mgN/L to only 10% at TAN of 6,000 mgN/L, while no obvious changes in carbohydrates were observed, confirming that protein degradation is more vulnerable to ammonium loading than carbohydrate degradation. Hence, the present high solid digester might have been under the sub-optimal status due to the higher initial TAN concentration (>3,000 mgN/L) in the start-up period. After ammonium addition from day 56, the increased protein content was in line with the changes of VS/TS which showed close correlation with R^2^ of 0.66, proving that excess ammonium was able to mediate and inhibit the pathway of protein degradation^32^.

When it comes to ammonium inhibition, previous studies mainly focused on the intrinsic links between environmental perturbation (e.g. ammonium stress) and methanogenesis^33^,^34^. However, it was of great possibility to attribute the deterioration of digestion performance to the decrease of bacterial diversity, as the obvious reduction of bacterial diversity was observed. *Tepidimicrobium* is reported as a rod-shaped bacteria belonging to *clostridia*. It is peptolytic and strictly nonsaccharolytic, and thus could grow organotrophically on a number of proteinaceous substrates^35^. *Proteiniborus*, similar to *Tepidimicrobium*, could also utilize proteins as fermentative substrates within a wide pH range from 6 to 8^36^. *Anaerobranca* is essentially proteolytic^37^,^38^. Prowe and Antranikian^39^ reported that the genus *Anaerobranca* comprises only a few species among anaerobic microorganisms that have the capacity to convert proteins to acetic acid as key final product at pH 6–10 and temperature 30–70 °C. The significant positive correlation with TAN elevation and the sharp rise on day 42 proved their responsibility in protein degradation particularly when ammonium concentration was high. The findings suggested that *Anaerobranca* experienced a relatively lower degradation rate than *Tepidimicrobium* and *Proteiniborus*.

As for methanogens, Karakashev *et al*.^40^ found that digesters with high-strength ammonium were often dominated by *Methanosarcinaceae*, which was in consistent with the results from the present study. *Methanosarcina* at species level, either with acetoclastic or hydrogenotrophic pathway, could be selected by the different environmental conditions such as ammonium stress^34^,^41^. De Vrieze *et al*.^34^ reported that a robust methanogenic process can be established based on the interactions between syntrophic acetate oxidization (SAO) and *Methanosarcina sp.* with acetoclastic methanogenesis by *Methanosarcina sp.* at low OLR and ammonium concentrations, and SAO coupled with hydrogenotrophic methanogenesis by *Methanosarcina sp.* at elevated OLR and ammonium concentrations. Thus, SAO could tolerate to high concentrations of ammonium^42^. Schnürer A and Nordberg Å^43^ clearly showed that at mesophilic temperature, ammonia is a strong selector for syntrophic acetate oxidation. The ^14^CO_2_/^14^CH_4_ ratio clearly increased with the elevation of ammonium concentration. The shift from the aceticlastic mechanism to the syntrophic pathway occurred distinctly as the ammonium concentration rose above 3,000 mgN/L. Meanwhile, SAO requires partnership of specific bacteria, of which most are members of the *Clostridia* class^42^. Schnürer A *et al*.^44^ found *Clostridium ultunense* sp. nov, as a mesophilic bacterium, oxidized acetate in syntrophic association with a hydrogenotrophic methanogenesis.

Karakashev *et al*.^41^ claimed that the acetate oxidation to H_2_/CO_2_ followed by the hydrogenotrophic pathway was dominant in the absence of *Methanosaetaceae*. In this study, CO_2_ content increased with the decrease of methane content from 64.8% in Phase II to 55.2% in Phase IV when TAN increased from day 56. Thus, SAO bacteria outweighed *Methanosarcina* in the acetate competition which probably further led to the enhancement of SAO pathway. In addition, the hydrolysis and degradation of organic matters especially proteins in our study was inhibited which eventually impaired the supply of available acetate.

The gradual shift of acetate utilization from methane production by acetotrophic methanogens to acetate oxidation by SAO bacteria is reported to be followed by the subsequent shift to SAO/hydrogenotrophic methanogenesis (SAO/HM)^25^,^26^. However, instead of the hydrogenotrophic methanogenesis, relatively stable acetoclastic methanogenic communities even after day 56 was incorporated with the promoted SAO pathway, which is thus suspected to be the microbial response to ammonium stress in this study and revealed the possibility of lagged synergistic effects from SAO and hydrogenotrophic methanogenesis. The combination of acetoclastic methanogens and the enhanced SAO bacteria could be thus the bio-marker of entry into the ‘suboptimal’ status of the digester. Therefore, the possible regulation of bacterial communities to ensure macromolecular degradation efficiency could be proposed to avoid methanogenesis adaption to hydrogenotropic pathway under ammonium stress and recover the optimal and stable performance for methane production.

In conclusion, we found that an inhibited steady-state was achieved along with the increase of VS/TS and protein content under 5000–6000 mgN/L TAN. The conducted pyrosequencing analysis revealed that bacterial communities were impacted significantly by ammonium stress. *Anaerobranca*, rather than *Tepidimicrobium* and *Proteiniborus*, was the dominant bacteria which played an important role in protein degradation under elevated TAN concentration. The NMDS results showed that TAN, instead of FAN, dictated the correlation with the microbial communities.

On the other hand, archaeal communities, with the constant dominance of *Methanosarcina*, showed no significant response. Thus, the high solid digester was limited by the hydrolysis step when ammonium increased. The dominant *Methanosarcina* and the increased carbon dioxide content from 35.2% (Phase II) to 44.8% (Phase IV) under ammonium stress suggested that, rather than the commonly acknowledged syntrophic acetate oxidation (SAO) with hydrogenotrophic methanogenesis, only SAO pathway was enhanced during the initial ‘ammonium inhibition’.

## Materials and Methods

### Characteristics of inoculum and feedstock

The inoculum was derived from an operated lab-scale mesophilic high solid anaerobic digester with dewatered sludge. Total solids (TS) and volatile solids/TS ratio (VS/TS) of inoculum were 13.6% and 46.6%, respectively. The feeding dewatered sludge was collected from a typical domestic wastewater treatment plant (WWTP) in Shanghai, China, with TS and VS/TS of 20.1% and 53.9%, respectively. Immediately before it was fed into the digester, the dewatered sludge was heated to 35 °C from a storage temperature of 4 °C.

### Operation of the high solid digester

The 9 L semi-continuous anaerobic digester equipped with a helix-type stirrer (HLZ-AR(DV)-9, Shanghai, China) was operated under mesophilic conditions (35 ± 1 °C) at a solid retention time (SRT) of 20 days, and was stirred at a speed of 60 rotations per minute (rpm) at a 10 min on and 10 min off interval.

For the start-up, 4.5 kg of inoculum sludge and 4.5 kg of dewatered sludge were both initially adjusted to TS of 15% and added to the digester. The biogas generation was recorded daily by a wet gas meter (LMF-1, Qingdao, China). TS of the daily feeding sludge was also adjusted to 15% by the addition of tap water. The digester was fed and withdrawn once a day.

The 110-day operation was divided into four phases: (I) the start-up period from day 1 to day 40; (II) the steady-state phase from day 41 to 55, during which TAN concentrations were spontaneously maintained at 3,000–3,500 mgN/L; (III) the ammonium adjusting period from day 56 to 70, when TAN concentration was increased iteratively to reach 5,000 mgN/L by mixing ammonium chloride into the feed; (IV) the ammonium stressed period from day 71 to 110 when TAN concentration was maintained within the range of 5,000–6,000 mgN/L.

### Analytical methods

During the experiment, samples were taken to analyze pH, biogas content, TS, VS/TS, volatile fatty acids (VFAs), alkalinity, TAN, FAN, proteins, carbohydrates, and lipids. pH was measured with a pH meter (S210, METTLER, Switzerland). Methane and carbon dioxide content was analyzed by a gas chromatograph (GC112A, INESA, China) with a thermal conductivity detector equipped with GDX-102 packed column (2 m*4 mm). TS, VS, alkalinity, and TAN were determined according to *Standard Methods for the Examination of Water and Wastewater*^45^. FAN concentrations were calculated from NH_4_^+ ^concentrations, temperature and pH^9^. To analyze VFAs, the supernatant of samples prepared by centrifugation at 13000 rpm for 20 min was passed through a microfiber filter (0.45 μm). The filtrate was then acidified to adjust pH to approximately 2.0 by the addition of formic acid, prior to VFAs being analyzed by a gas chromatograph (GC) (2010 plus, Shimadzu, Japan) with flame ionization detector (FID). The determination of proteins was achieved by the multiplication of Kjeldahl nitrogen by the coefficient of 6.25 (Kjeltec 9860, Hanon, China). The content of lipids in samples was analyzed by Soxhlet extraction apparatus (Soxhlet 2050, Foss, Denmark), and that of carbohydrates in samples with 2.5 N HCl pretreatment for 3 h at 100 °C was determined via the anthrone method, with glucose as the standard^46^. The pretreatment efficiency further assessed the solubilization of carbohydrate using Fourier Transform Infrared Spectroscopy (FTIR) analysis. The samples before and after HCl treatment were collected and freeze-dried. The transmittance of them under all detected wavenumbers were compared, which indicated that the HCl pretreatment increased the transmittance including the typical functional groups for carbohydrate of OH stretching (3000–3600 cm^−1^), C-H stretching (2860–2970 cm^−1^), and C = O stretching (1510–1560 cm^−1^), and confirmed the feasibility of the method (Figure S4)^47^. All the analyses were conducted in triplicate.

### Microbial population analyses

The DNA samples were extracted using the PowerSoil DNA Isolation Kit (MO BIO, USA), with bacterial and archaeal populations investigated using pyrosequencing analysis (Miseq4000, Illumina) after polymerase chain reaction (PCR). The PCR product concentrations were assessed using a QuantiFLuorTM system (Promega), and purified using an AxyPrep DNA gel extraction kit (AXYGEN, USA). The quality of the purified PCR products was verified using gel electrophoresis. Barcode sequences were attached to the 338 f(5′-ACTCCTACGGGAGGCAGCA-3′)/806r(5′-GGACTACHVGGGTWTCTAAT-3′) bacterial primer set^48^ and the 344 f(5′-ACGGGGYGCAGCAGGCGCGA-3′)/915r(5′-GTGCTCCCCCGCCAATTCCT-3′) archaeal primer set^49^ following the guideline provided by Illumina Company (San Diego, California, USA). The obtained data was analyzed using QIIME 1.8 pipeline by selecting sequences with an average quality value of 25, longer than 200 bp, containing short homopolymers (<8 bp) and less than two primer mismatches^50^. The sequences have been deposited into the NCBI short read archive (SRA) under the accession number SRR 3068598 for bacteria and SRR 3073815 for archaea.

### Quantitative PCR

The real-time PCR was conducted using the Sybergreen system. The target methanogen was determined according to the relative abundance results obtained from pyrosequencing analysis. The family-level specific primer set with forward primer (5′-GAAACCGYGATAAGGGGA-3′) and reverse primer (5′-TAGCGARCATCGTTTACG-3′) was selected to detect the changes of *Methanosarcinaceae* (Msc) in this study^51^. The amplification buffer consisted of 10 μL SYBR *Premix Ex Taq* II (Tli RNaseH Plus) (2x) (TaKaRa, Japan), 0.8 μL forward primer (10 μM), 0.8 μL reverse primer (10 μM), 2 μL template DNA and PCR-grade sterile water, to a final volume of 20 μL. All experiments were triplicated.

Samples were performed real time PCR analysis with 40 cycles of amplification in a Thermal Cycler Dice^TM^ Real Time System III (TaKaRa, Japan) under the following thermal cycling conditions: firstly, samples were preheated at 95 °C for 30 s; subsequently, 40 cycles of 15 s 95 °C, 20 s 55.1 °C and 40 s 72 °C were employed. Detection of fluorescence of the product was carried out after the last step of each cycle.

### Statistical analysis

The errors presented in the text are 95% confidence intervals in mean based on two-tailed t-tests of the measured or calculated values including TAN, FAN, biogas yield, pH, volatile solid reduction, and alkalinity. The copies of *Methanosarcina* in each phase were presented in their mean values with standard deviations. Analysis of Variance (ANOVA) was used to determine if the difference in individual organic component after day 56 was significant. The pyrosequencing data of the bacterial communities was transformed into a quantitative matrix, and further analyzed by non-metric multidimensional scaling (NMDS), as NMDS analysis is usually considered the most effective ordination method for ecological community data^29^. NMDS ordination, as well as the joint-plot analysis with parameter interpretation (i.e., pH, biogas yield, alkalinity, TAN, FAN, VFA, acetate), was conducted based on Sorensen (Bray-Curtis) distance in R with the *Vegan* package in the study^52^,^53^. The effect of each factor on the ordination of community profiles is presented with the length of arrows which is proportional to the magnitude of the correlation^54^.

## Additional Information

**How to cite this article**: Dai, X. *et al*. Metabolic adaptation of microbial communities to ammonium stress in a high solid anaerobic digester with dewatered sludge. *Sci. Rep.* **6**, 28193; doi: 10.1038/srep28193 (2016).

## Supplementary Material

Supplementary Information

## Figures and Tables

**Table 1:** Summarized operational parameters during the four phases (with 95% confidence intervals).

Parameters	Phase I (day 1–40 )	Phase II (day 41–55 )	Phase III (day 56–70 )	Phase IV (day 71–110 )
TAN (mgN/L)	4209 ± 510	3860 ± 2080	4287 ± 667	5443 ± 247
Biogas (L/day)	9.8 ± 1.8	11.9 ± 0.3	10.8 ± 0.5	10.4 ± 0.2
pH	7.98 ± 0.07	7.94 ± 0.02	7.98 ± 0.03	7.94 ± 0.01
VS reduction (%)	23.5 ± 3.3	31.6 ± 0.9	27.3 ± 3.5	21.3 ± 1.5
