# Supplementary material for: Metabolic adaptation of microbial communities to ammonium stress in a high solid anaerobic digester with dewatered sludge
Source: Sci Rep. 2016 Jun 17;6:28193. doi: 10.1038/srep28193 (PMC4911566; doi:10.1038/srep28193)
Supplement: Supplementary Information [file srep28193-s1.pdf]

Metabolic adaptation of microbial communities to ammonium stress in a high solid anaerobic digester with dewatered sludge

Xiaohu Dai[1, 2], Han Yan[1, 2], Ning Li[1, *], Jin He[1], Yueling Ding[1], Lingling Dai[1], Bin Dong[1]

1. State Key Laboratory of Pollution Control and Resource Reuse, College of Environmental Science and Engineering, Tongji University, 1239 Siping Road,

Shanghai 200092, PR China

2. These authors contributed equally in this work

* Correspondence and requests for materials should be addressed to Ning Li (lining@tongji.edu.cn)

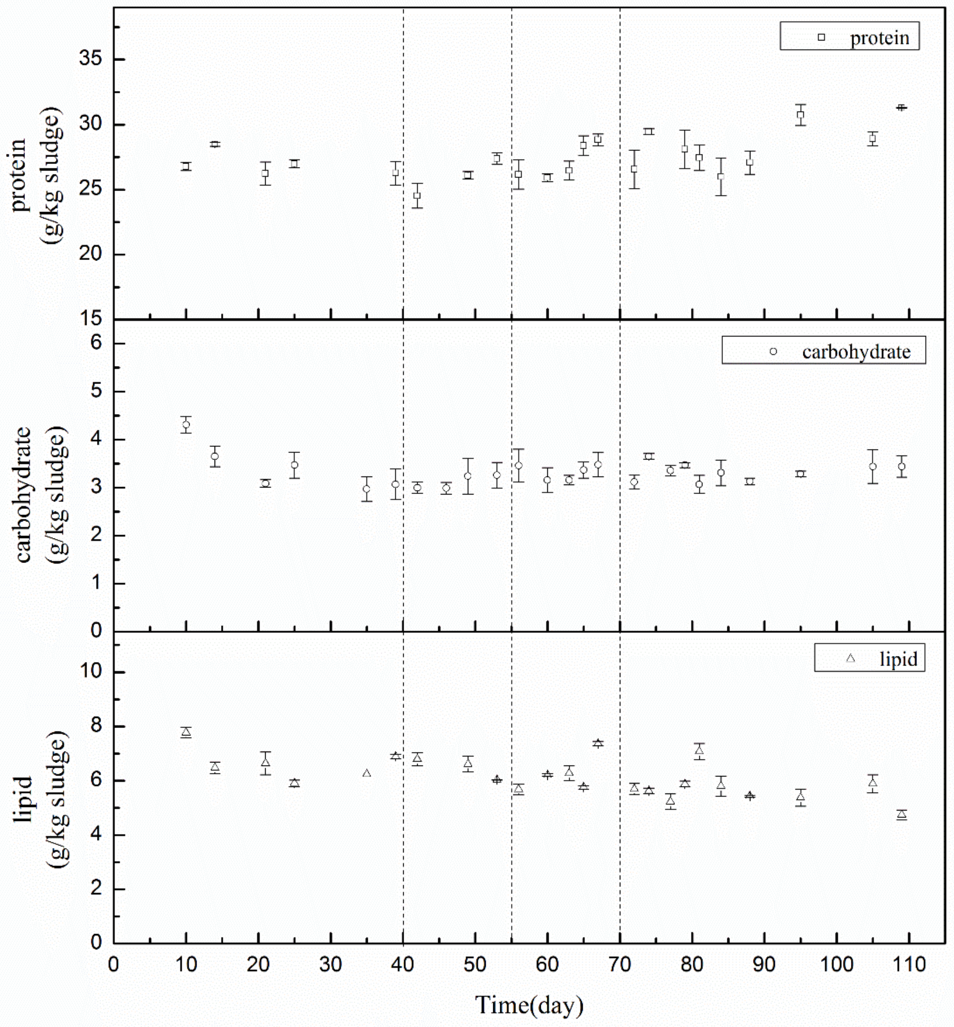

Figure S1. Changes in relative abundance of bacteria communities during the operation

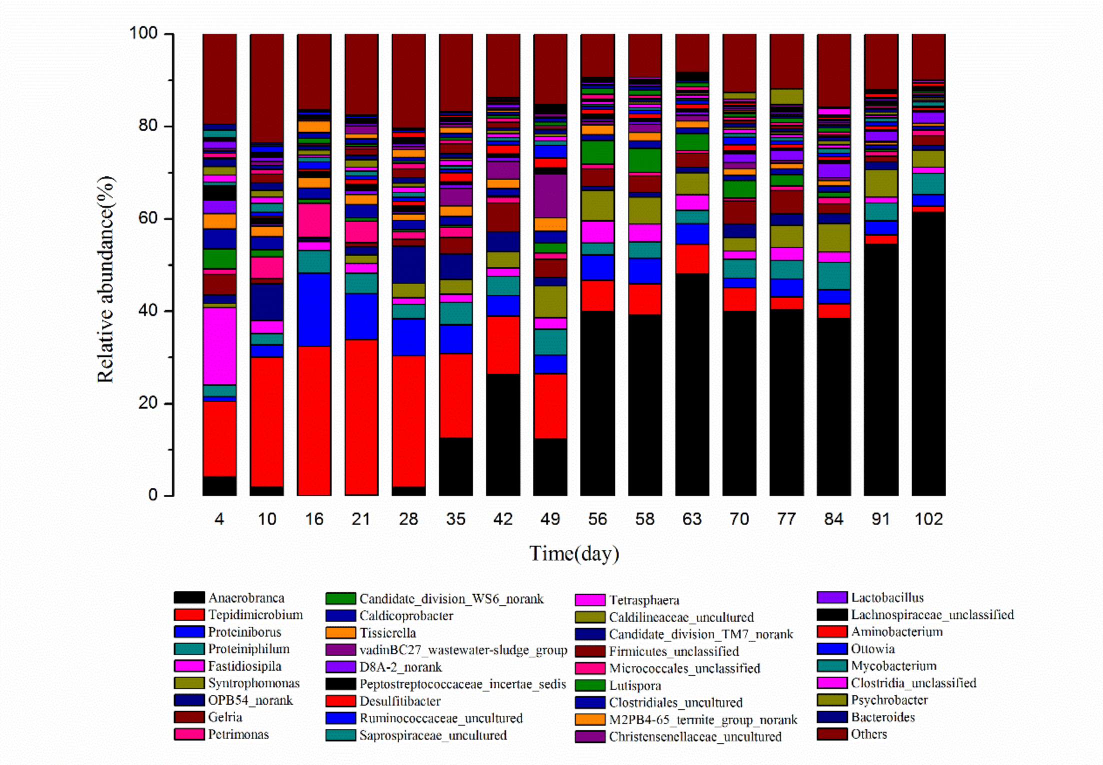

Figure S2. Variations of protein, carbohydrate and lipid during the operation

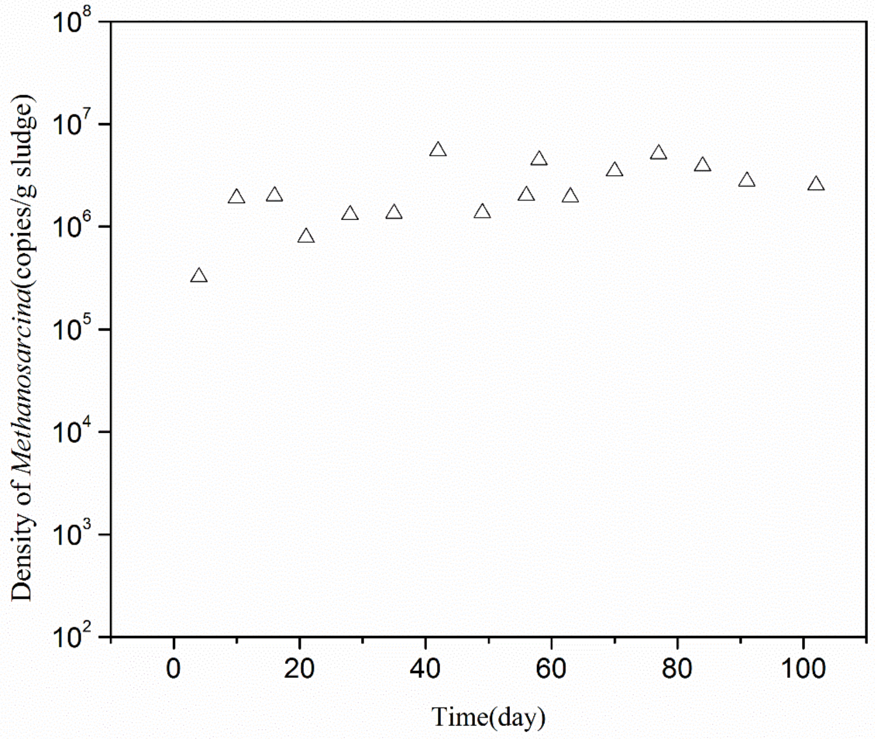

Figure S3. Changes of *Methanosarcina* density during the operation

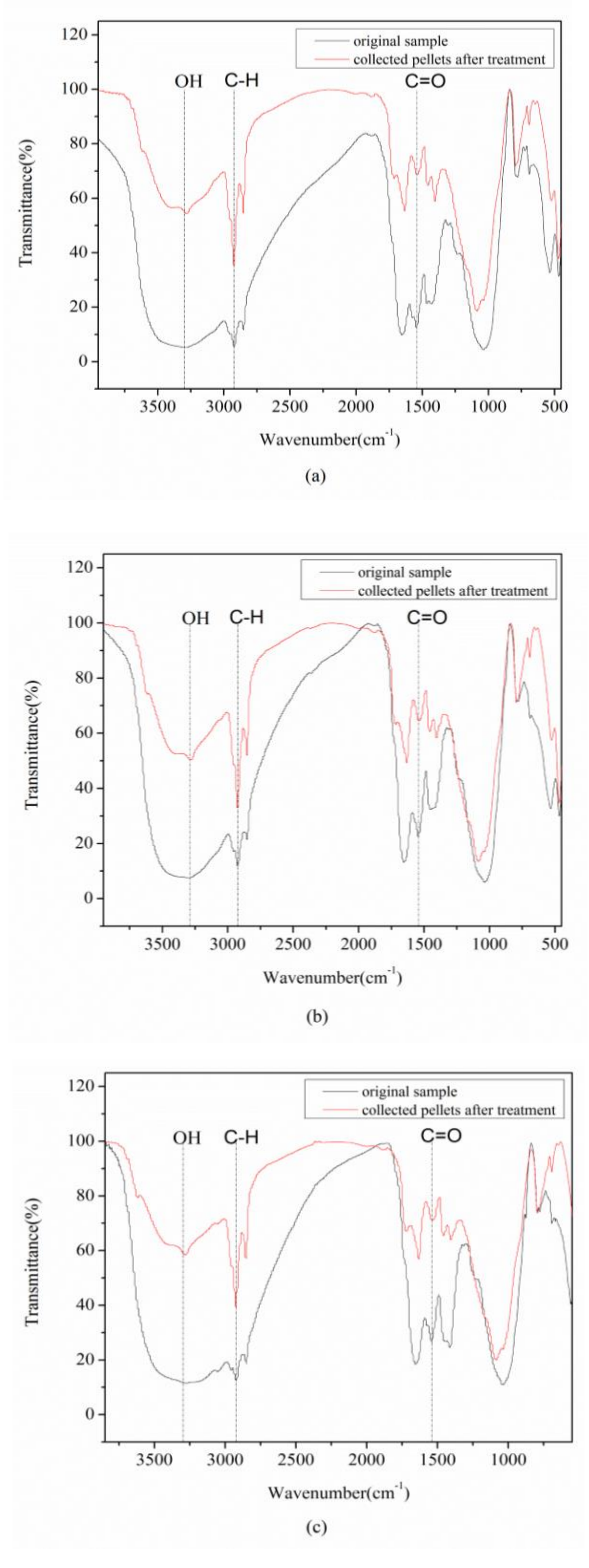

Figure S4. FTIR spectrograms of collected samples before and after HCl treatment (a) Sample from day 25; (b) Sample from day 56; (c) Sample from day 84.
